# Three New Acrylic Acid Derivatives from *Achillea mellifolium* as Potential Inhibitors of Urease from Jack Bean and *α*-Glucosidase from *Saccharomyces cerevisiae*

**DOI:** 10.3390/molecules27155004

**Published:** 2022-08-06

**Authors:** Umar Farooq, Sara Khan, Sadia Naz, Tanveer A. Wani, Syed Majid Bukhari, Abullahi Tunde Aborode, Sohail Anjum Shahzad, Seema Zargar

**Affiliations:** 1 Department of Chemistry, COMSATS University Islamabad, Abbottabad Campus, Abbottabad 22060, KPK, Pakistan; 2 Department of Pharmaceutical Chemistry, College of Pharmacy, King Saud University, Riyadh 11451, Saudi Arabia; 3 Department of Chemistry, Mississippi State University, Starkville, MS 39762, USA; 4 Department of Biochemistry, College of Science, King Saud University, Riyadh 11451, Saudi Arabia

**Keywords:** acrylic acid, spectroscopic analysis, *Achillea mellifolium*, enzyme inhibition, molecular docking, MD simulations, MMPBSA

## Abstract

(1) Background: *Achillea mellifolium* belongs to a highly reputed family of medicinal plants, with plant extract being used as medicine in indigenous system. However, limited data is available regarding the exploitation of the medicinal potential of isolated pure compounds from this family; (2) Methods: A whole plant extract was partitioned into fractions and on the basis of biological activity, an ethyl acetate fraction was selected for isolation of pure compounds. Isolated compounds were characterized using different spectroscopic techniques. The compounds isolated from this study were tested for their medicinal potential using in-vitro enzyme assay, coupled with in-silico studies; (3) Results: Three new acrylic acid derivatives (**1**–**3**) have been isolated from the ethyl acetate fraction of *Achillea mellifolium*. The characterization of these compounds (**1**–**3**) was carried out using UV/Vis, FT-IR, 1D and 2D-NMR spectroscopy (^1^H-NMR, ^13^C-NMR, HMBC, NOESY) and mass spectrometry. These acrylic acid derivatives were further evaluated for their enzyme inhibition potential against urease from jack bean and *α* glucosidase from *Saccharomyces cerevisiae,* using both in-silico and in-vitro approaches. In-vitro studies showed that compound **3** has the highest inhibition against urease enzyme (IC_50_ =10.46 ± 0.03 μΜ), followed by compound **1** and compound **2** with percent inhibition and IC_50_ value of 16.87 ± 0.02 c and 13.71 ± 0.07 μΜ, respectively, compared to the standard (thiourea-IC_50_ = 21.5 ± 0.01 μΜ). The investigated IC_50_ value of compound 3 against the urease enzyme is two times lower compared to thiourea, suggesting that this compound is twice as active compared to the standard drug. On the other hand, all three compounds (**1**–**3**) revealed mild inhibition potential against *α*-glucosidase. In-silico molecular docking studies, in combination with MD simulations and free energy, calculations were also performed to rationalize their time evolved mode of interaction inside the active pocket. Binding energies were computed using a MMPBSA approach, and the role of individual residues to overall binding of the inhibitors inside the active pockets were also computed; (4) Conclusions: Together, these studies confirm the inhibitory potential of isolated acrylic acid derivatives against both urease and *α*-glucosidase enzymes; however, their inhibition potential is better for urease enzyme even when compared to the standard.

## 1. Introduction

Natural products are an appealing source of therapeutic compounds, and a substantial amount of research is focused on the isolation of compounds from natural product extracts, such as plants with indigenous importance. In-lab approaches for screening enzyme inhibitors from natural product extracts are based on spectroscopy, which frequently determines enzyme activity using specially designed enzyme assays. *Achillea millefolium,* locally named as Kangi Jari or Kala Chahu, is a perennial herb belonging to the Asteraceae family, also referred to as a composite family. Asteraceae is the largest angiosperm family, comprised of over 1620 genera and 23,000 species. Achillea is a genus of 115 species that are mostly perennial herbs. *Achillea millefolium* is native to temperate and sub-tropical regions of the world and grows well in meadows [1,2].

*Achillea millefolium* is an ornamental plant with a sweet fragrance. Traditionally, it has been used as an astringent, an analgesic, as an antispasmodic agent, vasoprotective agent and for healing wounds [3,4,5]. It has also been used for treatment of digestive disorders, high blood pressure, menstruation irregularities and as a diuretic agent [6,7,8]. The oil of *Achillea mellifolium* has been reported as effective for the cure of flu, cold, gastrointestinal disorders, and toothache [9]. Urinary tract infections and kidney stones have also been treated with its aqueous extract.

Georgieva et al. reported anti-oxidant activity of water extract from *Achillea millefolium* [10,11]. In addition, antinociceptive, anti-inflammatory, antimicrobial, antitumor, anti-hepatoma and gastroprotective activities of *Achillea millefolium* have been reported previously [12,13,14,15,16,17]. Previously, the methanolic and aqueous extract of *Achillea mellifolium* has been extensively studied for its anti-oxidant activity [18].

Owing to its huge therapeutic potential, extensive phytochemical studies of *Achillea mellifolium* have previously been reported by various groups. Miller and co-worker reported achilleine, while Tozyo et al. identified achimillic acids A, B and C from extracts of *Achillea mellifolium* [15,19]. Similarly, azulene, chamazulene, rutin, artemetin, coumarins and 1,8-cineole have previously been reported [20,21,22,23]. In addition to these sequiterpenes, flavonoids such as luteolin and apigenin have also been identified [24,25].

Anne Orav et al. reported presence of sabinene, *β*-pinene, linalool, camphor etc., from essential oils of *Achillea mellifolium* [26]. In-vitro studies coupled with computational techniques for drug discovery have been shown to be useful in guiding the discovery of compounds capable of binding efficiently to biological targets, such as proteins, throughout time and in diverse studies. These interactions can be utilized to identify antibiotics, antimicrobial agents, and modulators in the human body.

Acetohydroxamic acid (Lithostat) is a prescription drug used to prevent the excessive build-up of ammonia in the urine of individuals with a persistent urea-splitting UTI. It inhibits urease by chelating with Ni ion, and is thus one of the most extensively researched chemicals as a potential therapy for ulcers produced by *H. pylori* [27,28,29,30]. Similarly, the strategy used for blood sugar regulation in diabetes depends on delaying the intestinal system’s absorption of glucose by blocking α-glucosidase enzymes, a crucial enzyme in the catabolism of complex carbohydrates. Given the significance of the enzyme in Diabetes Mellitus, several research efforts have been made to develop potential drugs to suppress the enzyme activity, as well as the fact that currently available treatments have limitations [31]. In a study, a variety of salicylic acid derivatives showed promising anti α-glucosidase activity, suggesting the viability of acid derivatives as prospective α-glucosidase inhibitors [32]. These intensify our efforts to find structural scaffolds that resist α-glucosidase, using a combination of in-vitro and in-silico methods. Keeping in mind the indigenous importance of this plant as a medicinal herb, together with the inhibitory potential of acid derivatives, in this study we have performed a phytochemical investigation of *Achillea mellifolium,* followed by exploration of the enzyme inhibition potential of isolated compounds (acrylic acid derivatives) against jack bean urease and *α*-glucosidase, using a combined in-vitro and in-silico approaches.

It is notable that all the isolated compounds (**1**–**3**) were more potent inhibitors of urease enzyme when compared to a standard inhibitor (thiourea). The best inhibition potential for urease was observed for compound **3** with IC_50_ value 10.46 ± 0.033 µM, while the other two compounds (**1**–**2**) also showed good inhibition potential. For *α*-glucosidase, mild inhibition tendency was revealed by all three acrylic acid derivatives. Furthermore, molecular docking studies, in combination with MD simulations and MMPBSA calculations, were carried out to unravel the time evolved behavior of compounds inside the active pocket, and the binding interaction pattern governed by key residues of target proteins (urease and *α*-glucosidase).

## 2. Results

### 2.1. Characterization of the Isolated Compounds

#### 2.1.1. Characterization of Compound **1**

**Figure 1:**
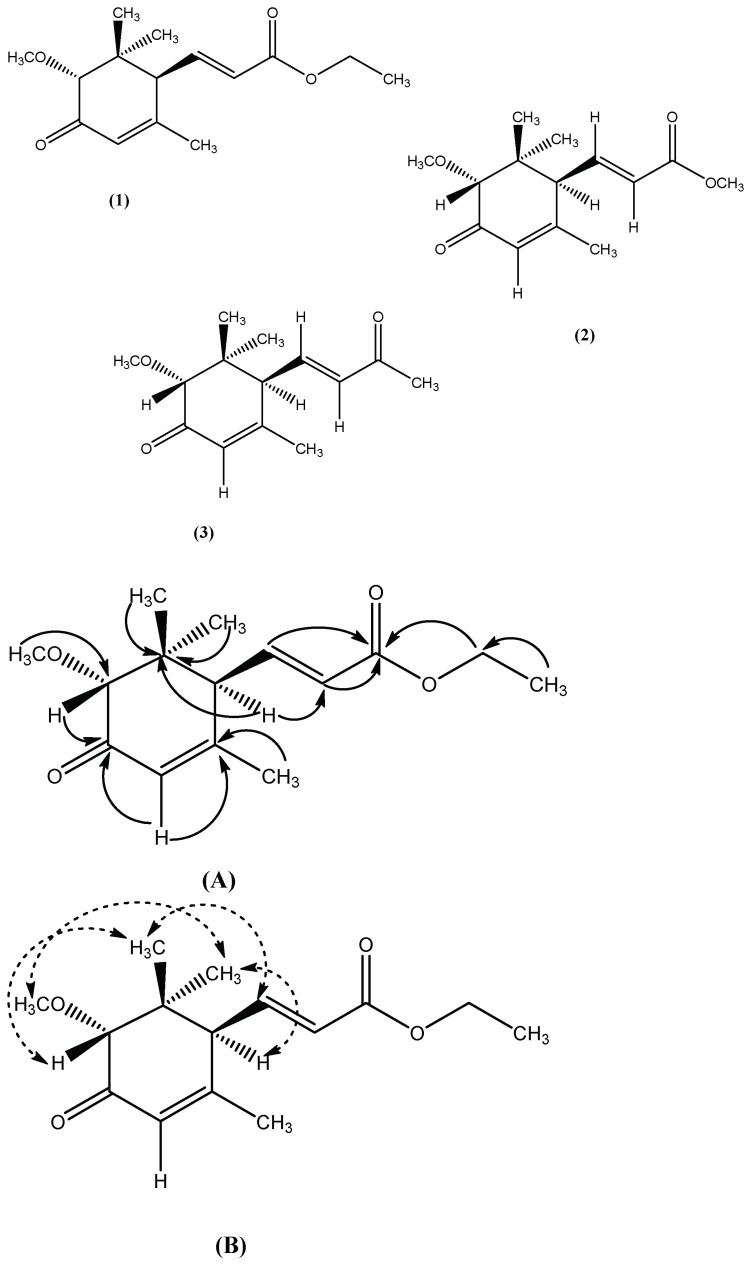
Chemical Structure of Compound **1**, **2** and **3**. (**A**). Important HMBC correlations (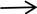), (**B**). NOE correlation (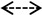).

A light-yellow gummy solid; UV (MeOH) λ_max_ 224 (4.01), 220 (3.8) nm; IR (KBr) υ_max_ 1672, 1730 and 2960 cm^−1^; EI-MS *m*/*z*: 266 [M]^+^ 250, 222, 208, 180, 172, 150, 124, 70 and 58; HR-EI-MS: *m*/*z* [M^+^] Calcd. 266.1518 for Mol. formula C_15_H_22_O_4_; Observed 266.1510; ${\left[\propto \right]}_{D}^{25}$ +121.26 (c = 0.81, CHCl_3_); ^1^H-NMR (500 MHz, CDCl_3_) δ (ppm): 3.80 (1H, s, H-2), 5.80 (1H, s, H-4), 2.81 (1H, d, *J* = 8.4 Hz, H-6), 6.30 (1H, dd, *J* = 15.8, 7.9 Hz, H-7), 5.98 (1H, d, *J* = 15.8 Hz, H-8), 3.78 (2H, q, *J* = 6.8 Hz, H-10), 1.35 (3H, t, *J* = 8.2 Hz, H-11), 0.98 (3H, s, H-12), 1.02 (3H, s, H-13), 3.42 (3H, s, H-14), 1.95 (3H, s, H-15); ^13^C-NMR (125 MHz, CDCl_3_) δ (ppm): 39.6 (C-1), 72.1 (C-2), 194.8 (C-3), 125.1 (C-4), 164.4 (C-5), 59.2 (C-6), 132.4 (C-7), 137.6 (C-8), 170.1 (C-9), 70.6 (C-10), 18.1 (C-11), 19.6 (C-12), 23.6 (C-13), 55.7 (C-14), 22.6 (C-15) (Figure 1).

#### 2.1.2. Characterization of Compound **2**

A gummy solid; UV (MeOH) λ_max_ 244 (4.01), 220 (3.8) nm; IR (KBr) υ_max_ 1680, 1740 and 2980 cm^−1^; EI-MS *m*/*z*: 252 [M]^+^ 220, 202, 236, 196, 174, 152, 122, 70 and 58; HR-EI-MS: *m*/*z* [M^+^] Calcd. 252.1362 for Mol. formula C_14_H_20_O_4_; Observed 252.1356; ${\left[\propto \right]}_{D}^{25}$ +104.86 (c = 0.68, CHCl_3_); ^1^H-NMR (500 MHz, CDCl_3_) δ (ppm): 3.74 (1H, s, H-2), 5.82 (1H, s, H-4), 2.78 (1H, d, *J* = 8.8 Hz, H-6), 6.28 (1H, dd, *J* = 16.4, 8.8 Hz, H-7), 5.92 (1H, d, *J* = 16.4 Hz, H-8), 3.85 (3H, s, H-10), 0.92 (3H, s, H-11), 1.08 (3H, s, H-12), 3.35 (3H, s, H-13), 1.90 (3H, s, H-14); ^13^C-NMR (125 MHz, CDCl_3_) δ (ppm): 38.2 (C-1), 70.8 (C-2), 192.1 (C-3), 126.7 (C-4), 167.1 (C-5), 58.1 (C-6), 134.8 (C-7), 141.1 (C-8), 172.1 (C-9), 56.1 (C-10), 19.8 (C-11), 23.4 (C-12), 57.9 (C-13), 22.6 (C-14) (Figure 1).

#### 2.1.3. Characterization of Compound **3**

A white solid; UV (MeOH) λ_max_ 238 (2.8) and 216 (3.7) nm; IR (KBr) υ_max_ 2920, 1720 and 1875 cm^−1^; EI-MS *m*/*z*: 236 [M]^+^ 218, 204, 174, 168, 150, 122, 70 and 56; HR-EI-MS: *m*/*z* [M^+^] Calcd. 236.1412 for Mol. formula C_14_H_20_O_3_; Observed 236.1406; ${\left[\propto \right]}_{D}^{25}$ +128.72 (c = 0.49, CHCl_3_); ^1^H-NMR (500 MHz, CDCl_3_) δ (ppm): 3.69 (1H, s, H-2), 5.85 (1H, s, H-4), 2.80 (1H, d, *J* = 8.0 Hz, H-6), 6.27 (1H, dd, *J* = 16.1, 8.5 Hz, H-7), 5.96 (1H, d, *J* = 15.7 Hz, H-8), 2.10 (3H, s, H-10), 0.97 (3H, s, H-11), 1.02 (3H, s, H-12), 3.40 (3H, s, H-13), 1.88 (3H, s, H-14); ^13^C-NMR (125 MHz, CDCl_3_) δ (ppm): 40.2 (C-1), 72.3 (C-2), 193.6 (C-3), 128.7 (C-4), 165.6 (C-5), 59.1 (C-6), 131.8 (C-7), 136.5 (C-8), 184.4 (C-9), 24.1 (C-10), 20.8 (C-11), 23.6 (C-12), 22.2 (C-13), 57.2 (C-14) (Figure 1).

The column chromatography of the ethyl acetate fraction resulted in isolation and purification of three new derivatives of acrylic acid, and their characterization was carried out using various spectroscopic techniques.

Compound **1** was isolated as a light-yellow colored gummy solid, having highest molecular ion peak at *m*/*z* 266.1510 in HR-EI-MS, suggesting the molecular formula of C_15_H_22_O_4_. The fragment peaks obtained were 250, 222, 208, 180, 172, 150, 124, 70 and 58. The absorption band in the UV spectrum were obtained at λ_max_ 224 (4.01), 220 (3.8), while IR spectrum confirmed presence of hydroxyl group, ester group and conjugated carbonyl moiety, as suggested by absorption band at υ_max_ 1672, 1730 and 2960 cm^−1^.

The ^1^H-NMR spectrum showed chemical shift values for three methyl groups at δH 0.98 (3H, s, H-12), 1.02 (3H, s, H-13) and 1.95 (3H, s, H-15) while two other singlets were also obtained at δH 3.80 (1H, s, H-2) and 5.80 (1H, s, H-4). Similarly, doublet was obtained at chemical shift value of 2.81 (1H, d, *J* = 8.4 Hz, H-6) and a doublet of doublet was obtained at δH 6.30 (1H, dd, *J* = 15.8, 7.9 Hz, H-7) while proton at position 8 also showed doublet at δH 5.98 (1H, d, *J* = 15.8 Hz, H-8).

In addition, a quartet for methylene protons at position 10 was obtained at δH 3.78 (2H, q, *J* = 6.8 Hz, H-10) and triplet at δH 1.35 (3H, t, *J* = 8.2 Hz, H-11) was also obtained, as given in Table 1. The singlet for methoxy protons was also observed at δH 3.42 (3H, s, H-14).

The ^13^C-NMR and DEPT spectrum showed the presence of fifteen (15) carbon atoms comprising of four methyl, one methylene, five methine and four quaternary and one methoxy carbon atom, as given in Table 2. The four methyl carbons resonated at δC 19.6 (C-12), 23.6 (C-13), 22.6 (C-15) and 18.1 (C-11), while methylene carbon was observed at chemical shift value of δC 70.6. The ^13^C-NMR spectrum revealed the presence of five methine carbon at δC 72.1 (C-2), 125.1 (C-4), 59.2 (C-6), 132.4 (C-7) and 137.6 (C-8). Similarly, four quaternary carbons resonated at δC 39.6 (C-1), 194.8 (C-3) and 170.1 (C-9) and signal for methoxy carbon was obtained at δC 55.7 (C-14).

The placement of substituents for compound **1** was done on the basis of HMBC and NOE spectra, as shown in Figure 1A and B. The methyl group protons at position 12 and 13 showed strong HMBC correlation with C-1, while other methyl protons at C-15 interacted with C-5, as revealed by HMBC spectrum. The methine proton at position 2 and 4 showed HMBC correlation with C-3 and C-5, respectively.

Strong HMBC correlation was shown by H-6 with C-1, C-7 and C-8, while proton at position 7 and 8 showed interaction with carbonyl carbon at position 9. Similarly, the placement of ethyl group substituent was also confirmed through their HMBC interaction patterns. In addition, the relative configuration of substituents was confirmed through Nuclear Overhauser Effect spectroscopy, and their interactions have been depicted in Figure 1. Finally, the structure of compound **1** was confirmed to be ethyl (E)-3-((1S,5R)-5-methoxy-2,6,6-trimethyl-4-oxocyclohex-2-en-1-yl) acrylate, as suggested by all spectral data, as well as literature comparison [33]. 

Compound **2** was obtained as a gummy solid, having molecular formula C_14_H_20_O_4_ as suggested by HR-EI-MS, with highest molecular ion peak at *m*/*z* 252.1356 and other fragment peaks were obtained at 220, 202, 236, 196, 174, 152, 122, 70 and 58. The UV spectrum showed two absorption bands at λmax 244 (4.01), 220 (3.8), while IR spectrum absorption band at υ_max_ 1680, 1740 and 2980 cm^−1^ suggesting the presence of hydroxyl group, ester group and conjugated carbonyl moiety, quite similar to compound 1.

The ^1^H-NMR spectrum showed the presence of two olefinic protons at δH 6.28 (1H, dd, *J* = 16.4, 8.8 Hz, H-7) and 5.92 (1H, d, *J* = 16.4 Hz, H-8), while two other protons gave singlets at δH 3.74 (1H, s, H-2) and 5.82 (1H, s, H-4), as shown in Table 1. The singlets for two methoxy groups appeared at δH 3.85 (3H, s, H-10) and 3.35 (3H, s, H-13). Similarly, singlets for protons of three methyl groups resonated at δH 0.92 (3H, s, H-11), 1.08 (3H, s, H-12) and 1.90 (3H, s, H-14). In addition, a doublet for one methine proton was also observed at δH 2.78 (1H, d, *J* = 8.8 Hz, H-6).

The ^13^C-NMR spectrum revealed the presence of fourteen carbon atoms including three methyl, five methine, four quaternary and two methoxy carbon. The chemical shift values for three methyl carbons were observed at δC 19.8 (C-11), 23.4 (C-12) and 22.6 (C-14), while two methoxy group carbons resonated at δC 56.1 (C-10) and 57.9 (C-13). The olefinic carbons showed chemical shift values of δC 126.7 (C-4), 134.8 (C-7) and 141.1 (C-8). The chemical shift value for quaternary carbon atoms appeared at 38.2 (C-1), 192.1 (C-3), 167.1 (C-5), and 172.1 (C-9). Similarly, two methine carbons were also observed at δC 70.8 (C-2) and 58.1 (C-6), as given in Table 2.

The HMBC, COSY and NOE spectra were used for accurate placement of substituents, as shown in Figure 1. The protons of methyl groups at position 11 and 12, and an olefinic proton (H-7), showed strong HMBC correlation with C-1 quite similar to compound **1**. Similarly, methoxy protons at position 10 and two olefinic protons i.e., H-7 and H-8, showed strong HMBC correlation with carbonyl carbon (C-9). In addition, methine protons (H-2 and H-4) showed HMBC interaction with carbonyl carbon C-3, while H-6 and H-7, along with methyl protons at position 14, revealed strong correlation with C-5.

The relative configuration of substituents was confirmed through NOE spectrum almost identical to compound **1**. All spectral data of compound **2,** and its comparison with literature [33] suggested it to be methyl (E)-3-((1S,5R)-5-methoxy-2,6,6-trimethyl-4-oxocyclohex-2-en-1-yl)acrylate; a derivative of acrylic acid.

Compound **3** was obtained as a white solid, having molecular ion peak at *m*/*z* 236.1406 by HR-EI-MS suggesting molecular formula C_14_H_20_O_3_. Other mass fragments obtained were *m*/*z* 218, 204, 174, 168, 150, 122, 70 and 56. The absorption band in UV spectrum were observed at λ_max_ 238 (2.8) and 216 (3.7), while IR spectrum confirmed the presence of functional groups such as hydroxyl group, ester group and conjugated carbonyl moiety, with absorption bands at 2920, 1720 and 1875, respectively, similar to compound **1** and **2**.

The ^1^H-NMR, as well as ^13^C-NMR spectra of compound **3,** were quite similar to compound **1** and **2**. The ^1^H-NMR spectrum showed singlets for four methyl protons at δH 0.97 (3H, s, H-11), 1.02 (3H, s, H-12), 1.88 (3H, s, H-14) and 2.10 (3H, s, H-10), while two olefinic protons resonated at δH 6.27 (1H, dd, *J* = 16.1, 8.5 Hz, H-7) and 5.96 (1H, d, *J* = 15.7 Hz, H-8), as shown in Table 1.

Similarly, methine proton i.e., H-2, H-4 and H-6, showed chemical shift value of δH 3.69 (1H, s, H-2), 5.85 (1H, s, H-4) and 2.80 (1H, d, *J* = 8.0 Hz, H-6), respectively. Another singlet was observed for methoxy protons at δH 3.40 (3H, s, H-13).

The ^13^C-NMR and DEPT spectrum of compound **3** showed the presence of fourteen carbon atoms, which was comprised of four methyl carbon, five methine, four quaternary and one methoxy carbon. The methyl carbon resonated at δC 20.8 (C-11), 23.6 (C-12), 22.2 (C-13) and 24.1 (C-10), and that of methoxy carbon at δC 57.2, as given in Table 2. The quaternary carbon showed signals at δC 40.2 (C-1), 193.6 (C-3), 165.6 (C-5) and 184.4 (C-9), while two olefinic methine carbons appeared at 131.8 (C-7) and 136.5 (C-8). In addition, two other methine carbons resonated at δC 128.7 (C-4) and 59.1 (C-6).

The HMBC and NOE spectra of compound **3** was almost similar to compound **1** and **2** and were quite helpful for placement of substituents at various positions, as shown in Figure 1. The only difference observed was HMBC correlation of methyl protons at position 10, with carbonyl carbon at C-9. All spectral data suggested the compound to be (4S,6R)-6-methoxy-3,5,5-trimethyl-4-((E)-3-oxobut-1-en-1-yl)cyclohex-2-en-1-one, which was also confirmed through literature comparison.

## 3. Discussion

Urease, a nickel-containing enzyme, has been reported as a potential target for discovery of potential antiulcer, as well as antigastric cancer drug candidates. The need for discovery of more potent and safe urease inhibitors prevails, as most of the previously reported inhibitors were toxic, with various side effects. The α-glucosidase is responsible for providing energy for the normal working of a human body. It has the tendency to catalyze carbohydrates in the body. High activity of α-glucosidase causes serious problem in human health due to an increased glucose level. The α-glucosidase inhibitors e.g., acarbose and miglitol, can regulate the glucose level in case of type-2 diabetes, along with their applications in the cure of many other diseases. This research will also focus on the identification of potent α-glucosidase inhibitors from plant sources. Bioactive compounds isolated from natural resources, particularly plants and their use as potential drug candidates is well-documented [34].

The isolated acrylic acid derivatives were tested for their in-vitro inhibition against jack bean urease and *α*-glucosidase. Thiourea (IC_50_ 21.5 ± 0.01 μΜ) was used as a standard inhibitor in urease assay, while acarbose for *α*-glucosidase. All three compounds revealed promising inhibitory potential against urease where compound **3** showed IC_50_ value 10.46 ± 0.03 µM, while IC_50_ value for compound **1** and **2** were 16.87 ± 0.02 µM and 13.71 ± 0.07 µM, respectively. The enzyme inhibition potential (i.e., IC_50_ values) and binding scores (i.e., kcal/mol) for both urease and *α*-glucosidase enzymes are given in Table 3. Results revealed that acrylic acid derivatives in the current study are more potent inhibitors with IC_50_ values, even better than a standard inhibitor i.e., Thiourea. To the best of our knowledge, urease inhibitory potential of acrylic acid analogues are being reported for the first time ever and will open new horizons for the discovery of new anti-ulcer agents. In the case of *α*-glucosidase, moderate to low inhibition potential was observed for all test compounds. All three compounds showed IC_50_ value higher than standard i.e., acarbose (IC_50_ = 287.1 ± 0.03 µM).

**Figure 2:**
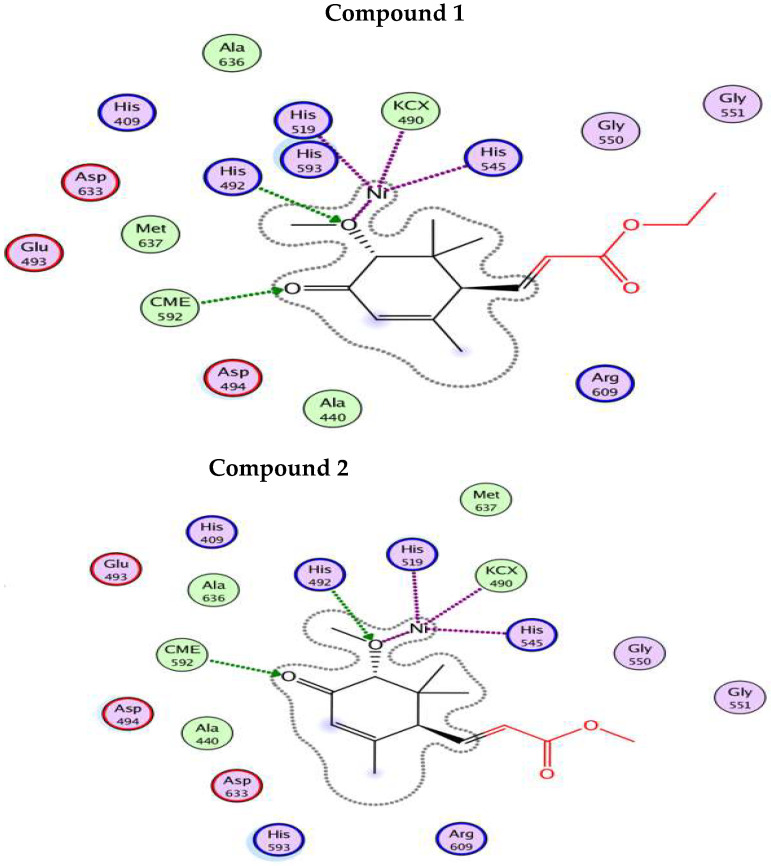

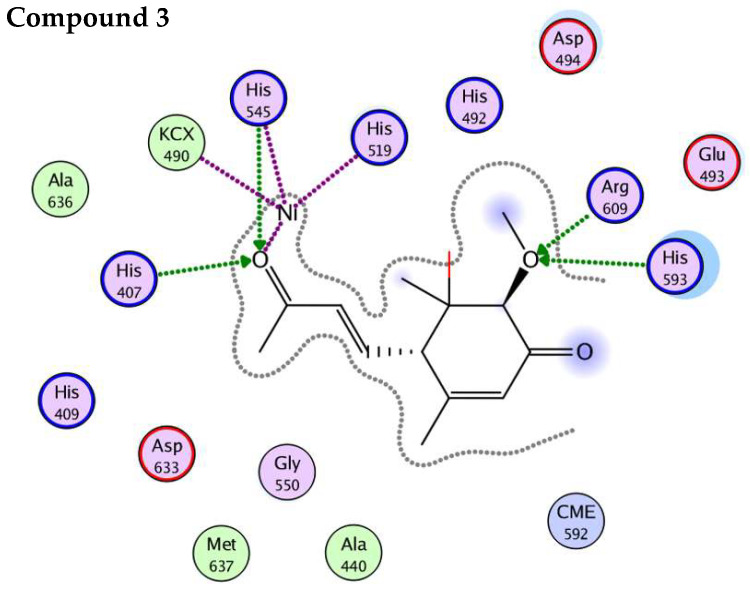
2D interaction plot of compounds (**1**–**3**) with active site residues of urease using LigX tools of MOE software. The three-letter amino acid code is assigned to each residue.

All these acrylic acid derivatives (**1**–**3**) showed good binding score within active site of urease where the best score (−9.831 Kcal/mol) was observed for compound **3,** compared to the standard with a binding energy of −3.332 Kcal/mol. It can safely be deduced from the results that compound **3** is three times potent compared to the standard drug. The ligand map reveals information, particularly the secondary contacts between the optimal docking pose and target proteins in term of strength of interactions, as shown in Figure 2. These include hydrogen bonding, steric interactions, and overlap interactions (violet circles) between active site residues and different portion of the inhibitors. The size of the circle on the atoms, which reflects the proportion of participation in steric hindrance, represents the strengths of the overlap contact. The main interactions observed were H-bond with His593, Arg609, His407, while His519 and His545 interacted through Ni atom (metal contact) within active site. Similarly, binding score observed for compound **1** and compound **2** were −7.011 and −7.224 kcal/mol, respectively. For both compound **1** and compound **2**, the main residues interacted through H-bond were His492, while His545 and His519 interacted through involvement of Ni atom.

**Figure 3:**
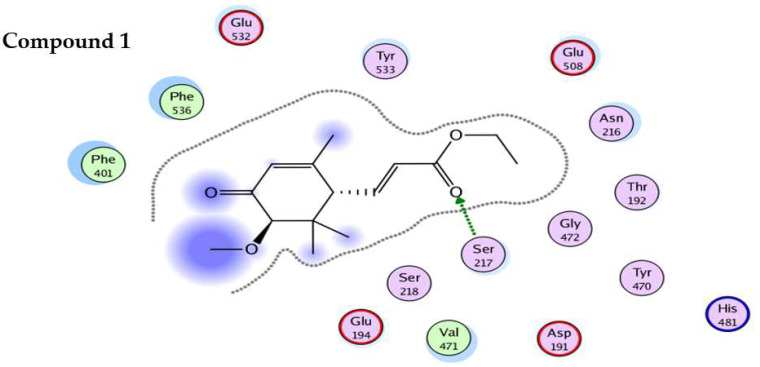

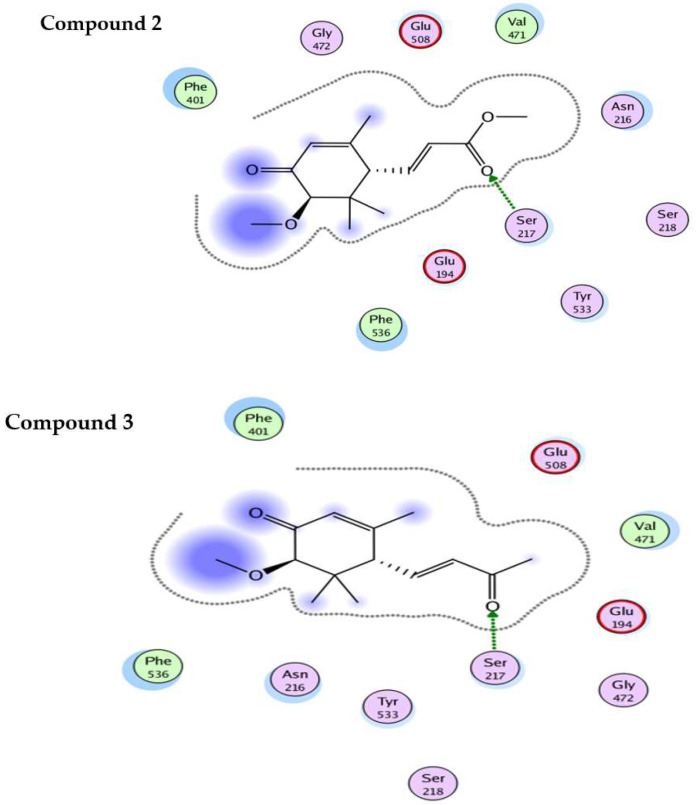
2D interaction plot of compounds (**1**–**3**) with active site residues of *α*-glucosidase using LigX tools of MOE software. The three-letter amino acid code is assigned to each residue.

Contrary to the standard (Acarbose-binding energy = −8.462 Kcal/mol), all these compounds (**1**–**3**) revealed low binding tendency against *α*-glucosidase, which is comparable to in-vitro enzyme inhibition potential. The only H-bonded interaction was observed for Ser217 and no other interaction, as shown in Figure 3. This good agreement between in-vitro and in-silico findings further strengthened the idea to use computational tools for in depth analysis of various biological activities.

Physical characteristics including HBA, HBD, total rotatable bonds, polar surface area, logP, and gastrointestinal absorption fit well into the criterion necessary for drug likeliness. Similarly, all the compounds strictly follow Lipinski rule, with minimum toxicity score of 4, as predicted by ProTox server (https://tox-new.charite.de/protox_II/index.php) (accessed on 4 May 2022) (Table 4).

An all-atom molecular dynamics (MD) simulation study was performed, with the intention of confirming the stability of the anticipated docked protein-inhibitor complexes. Employing this approach also provides useful information on the ligand’s dynamic behavior, as well as key binding interactions of inhibitor with critical catalytic site residues. Thus, the predicted protein-ligand complexes were subjected to 50 ns all-atom MD simulations.

**Figure 4:**
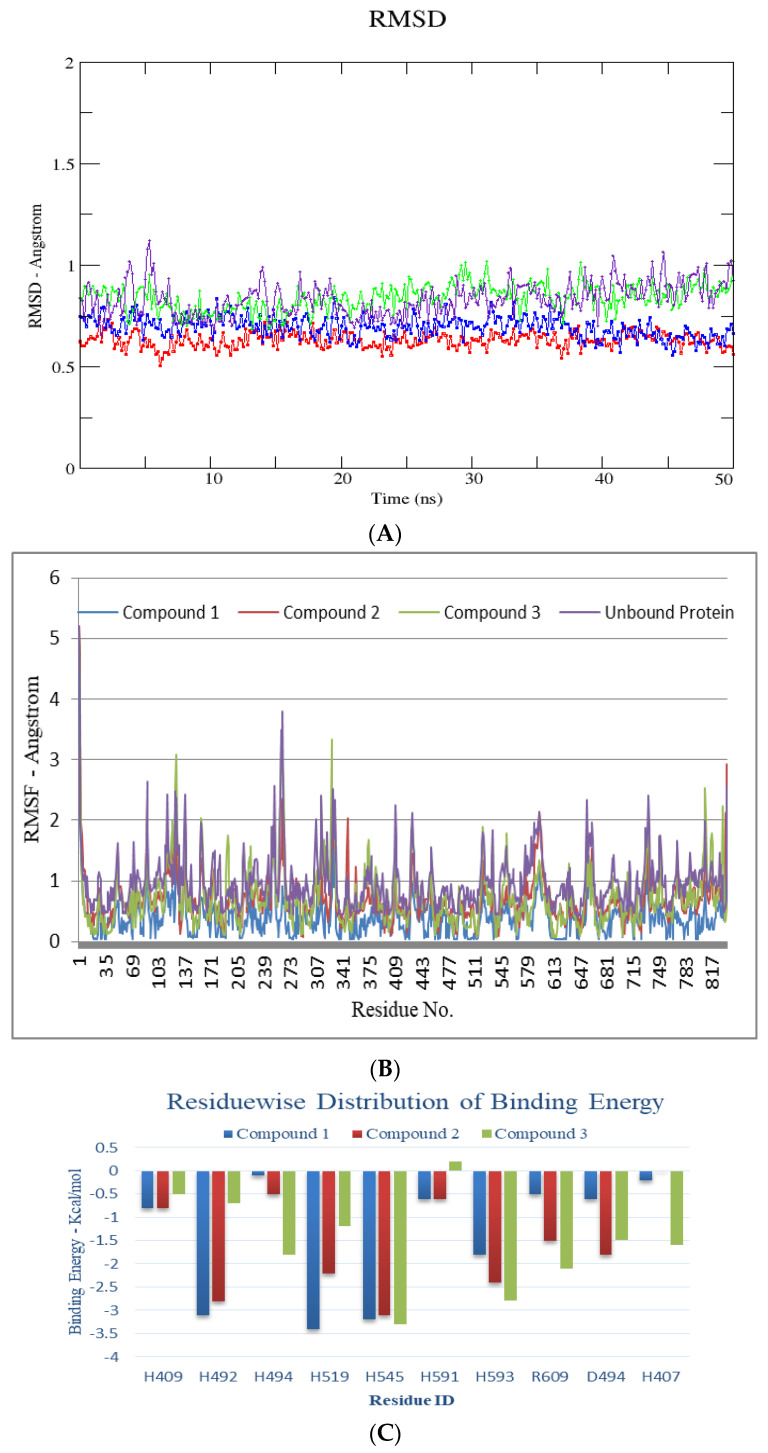

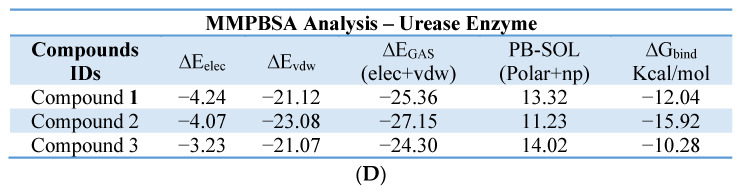
RMSD (all the atoms) (**A**) and per residue RMSF plot (**B**) of urease enzyme (PDB ID: 4GOA) in free and bound form with respect to initial minimized structures. Protein ligand complexes are color coded as Compound **1** (blue), Compound **2** (red), Compound **3** (green), Free protein (purple). (**C**) Major residues contributing towards protein ligand interactions. (All values are given in Kcal/mol) (**D**) Free energy of binding ΔG_bind_ for all compounds (**1**, **2** and **3**) using MMPBSA approach.

**Figure 5:**
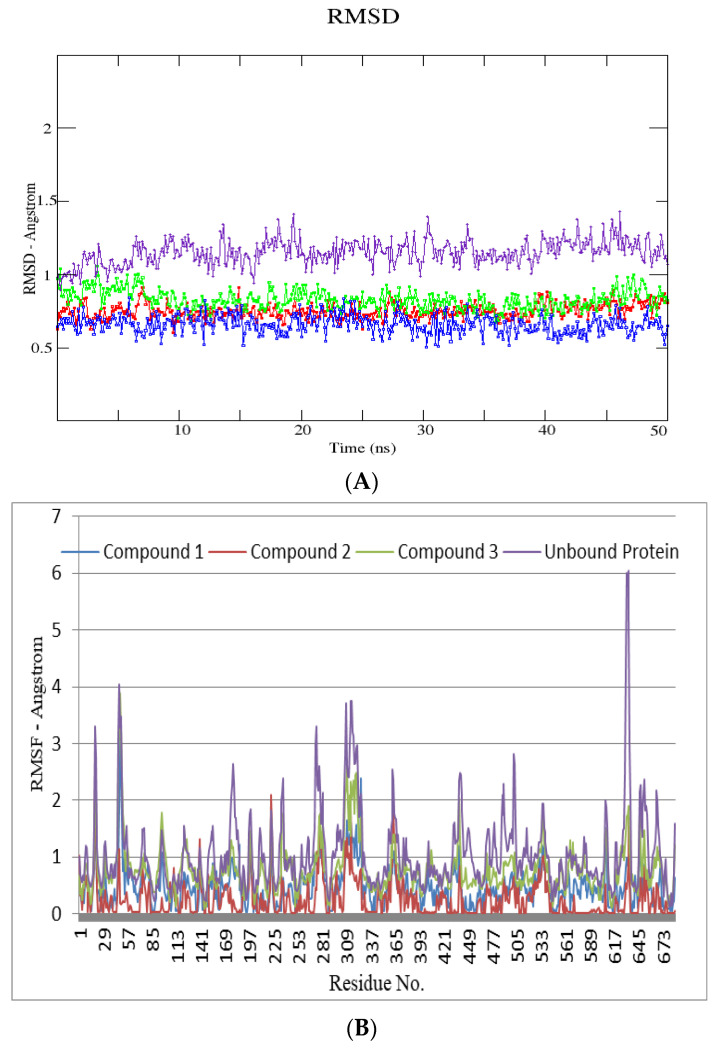

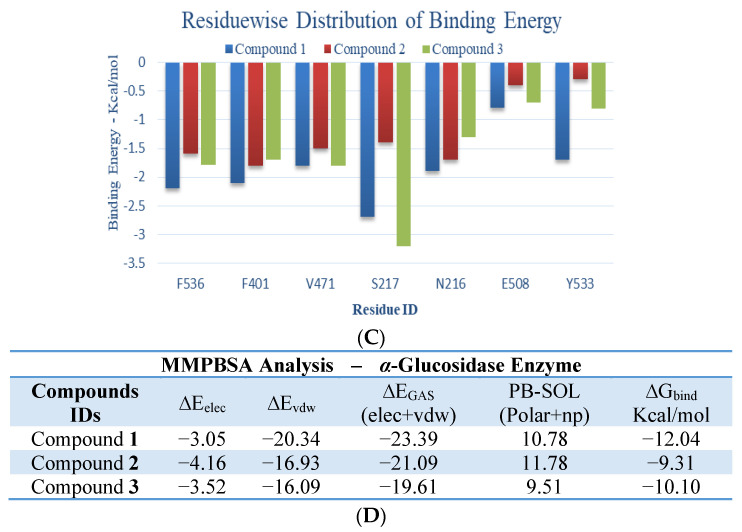
All atoms RMSD (**A**) and per residue RMSF plot (**B**) of *α*-glucosidase enzyme (PDB ID: 2JKE) in free and bound form with respect to initial minimized structures. Protein ligand complexes are color coded as Compound **1** (blue), Compound **2** (red), Compound **3** (green), Free protein (purple). (**C**) Major residues contributing towards protein ligand interactions. (All values are given in Kcal/mol) (**D**) Free energy of binding ΔG_bind_ for all compounds (**1**, **2** and **3**) using MMPBSA approach.

An all-atom molecular dynamics (MD) simulation analysis was done with the intention of evaluating the stability of the predicted docked ligand-protein complexes. Adopting such a study would provide useful information about the dynamic behavior of both the ligand bound and free protein, as well as assess the ligand’s key binding interactions with important catalytic site residues. As a result, the predicted protein complexes and their apo forms (urease-4GOA and α-glucosidase-2JKE) were subjected to within 100-ns all-atom MD simulations. The presences of ligand had little effect on protein dynamics, resulting in an average RMSD of 0.5 and 0.9 Å for ligand bound and free protein, respectively for urease (Figure 4). In the case of α-glucosidase, however, there is a significant difference in the amplitude of fluctuation between free protein (~1.3 Å) and complexes (0.7 Å), implying that an empty active site causes substantial fluctuations, but complexed state confirms a slight rigid behavior induced by the ligand upon binding to the active site residues (Figure 5). RMSF studies were used during MD simulations to uncover mean per-residue fluctuations. The alpha helices and beta sheets of urease are the most stable portions, whereas the loops connecting the domains are the most elastic region in both complexed and free protein. The urease enzyme’s loops encircling the catalytic site only showed minor fluctuations, indicating their role in maintaining the active pocket. During the simulation, the catalytic residues, primarily histidines, remained extremely stable. For α-glucosidase protein, despite the different flexibility pattern within the various loop sections in both complexed and free states, the complexed protein structure proved to be relatively stable, with a maximum RMSF of <2 Å, except the loop region, that showed higher amplitude fluctuations.

All of the compounds appear to be potent inhibitors for urease enzyme and their binding affinities, as revealed by the MMPBSA study, which confirms good binding of these compounds inside the active pocket (competitive inhibition), facilitated by hydrogen bond and hydrophobic interactions. In the case of urease, all the compounds bind close to nickel ions, in a manner analogous to the urea or thiourea fragment being complexed by nickel (II) ion. Visual analysis of the trajectories obtained also reveals that binding of acrylic acid derivatives to target protein, also demonstrated that these compounds effectively occupy active pockets of the target protein, firmly attaching the helix-turn-helix motif as a lid over the active-site cavity. This inhibits the urease active-site cavity flap from closing, therefore inhibiting the enzyme’s action. Interaction pattern reveals that binding of the compounds to the active pocket results in the formation of Ni-O electrostatic bond, whereby Ni bound oxygen of the compounds **1**–**2** forms h-bonds with His492 and to His407 in compound **3**, the latter being protonated in the presence of charged residue Asp633 in the proximity. Ni atom shows additional electrostatic interactions with His519 and His545 in compound **1**, **2** and **3**. Apart from this, oxygen atom of the methoxy group attached to position 3 on cyclohexene forms close contact with Arg609 and His593. The H-bonding network between the active site residues and compound **1**–**3** has clearly established the protonation state of the bound inhibitors, clarifying, and complementing, the pH dependency of the inhibition kinetics in most cases. The neutral form of compounds **1**–**3** is also well recognized, and the H-bond network just emphasizes the importance of surrounding residues in stabilizing a unique binding mechanism of oxygen to the metal center. Also, Michael acceptors (*α*,*β*-unsaturated ketones, aldehydes, esters, amides, and nitro compounds) are good urease inhibitors (Figure 4). The presence of the Michael group (*α*,*β*-unsaturated carbonyls) facilitates orientation, which aids maximum inhibitor binding inside the active pocket. Based on the acrylic acid functionality, the current structure provides vital clues for drug design of more effective urease inhibitors [29]. For α-glucosidase, the binding of compounds **1**–**3** is facilitated by multiple charged residues such as Glu508, Glu532 and Glu194, with Asn216 surrounding the active site, facilitating the retention of the compounds inside the active pocket. Similarly, Ser217 interacts via electrostatic interaction with compounds **1**–**3** (Figure 5). However, the interaction with active site Ca^+2^ ion was short-lived, making these compounds moderately active for this enzyme, also confirmed by in-vitro studies.

## 4. Materials and Methods

### 4.1. General Experimental Techniques

The double focusing Varian MAT-312 spectrometer was employed for EI-MS and HR-EI-MS analysis, while 1H-NMR and 13C-NMR spectra were recorded using Bruker AMX-500 MHz spectrometer. Silica gel (E. Merck 230–400 mesh and 70–230 mesh) was used for column chromatography and precoated silica gel plates were employed for TLC analysis.

The values of scalar coupling and chemical shift were reported in Hertz (Hz) and ppm, respectively. The IR spectra of compounds were recorded using Hitachi JASCO-320-A, and Hitachi UV-3200 spectrophotometer was employed to record UV spectra. The detection of UV active compounds was conducted through ceric sulphate in 10% H_2_SO_4_ solution. MOE software was used for molecular docking studies.

### 4.2. Plant Material

The whole plant of *Achillea mellifolium* (4 Kg) was collected from different areas of Abbottabad district, KPK, Pakistan, in July 2018 and a voucher specimen (No. 9816) has been deposited in herbarium at the Department of Botany Postgraduate College, Abbottabad, Pakistan.

### 4.3. Extraction and Isolation

The shade dried plant was ground into fine powder and soaked in methanol for two weeks at room temperature; filtrate was obtained after filtration. The filtrate was then subjected to vacuum rotary evaporator to obtain a crude extract (410 g). The fractionation of this crude extract resulted in four different fractions i.e., n-hexane (136 g), chloroform (90 g), ethyl acetate (105 g) and n-butanol (78 g).

On the basis of TLC analysis, the ethyl acetate fraction was selected by column chromatography for further investigation. Column chromatography was done using n-hexane as gradient of ethyl acetate to 100%, followed by methanol. Re-column chromatography resulted in 14 sub-fractions (A1–A14); out of these sub-fractions (A6–A13) were again subjected to column chromatography, resulting in isolation of compound 1 (14 mg) and compound **2** (13 mg) at polarity of n-hexane: ethyl acetate (35:65) and (40:60), respectively. Similarly, compound **3** (16 mg) was purified from sub-fractions (A2–A4) at polarity at n-hexane: ethyl acetate (30:70).

### 4.4. Enzyme Inhibition Studies

#### 4.4.1. Urease Inhibition Assay

The assay mixture, containing 50 μL (2 mg/mL) of jack-bean urease (purchased from Sigma Aldrich) and 100 μL of different concentration of test compounds, which were previously dissolved in ethanol 20%, was added to 850 μL of 25 mM urea and pre-incubated for 0.5 h in a water bath at 37 °C. The urease reaction was stopped after 30 min incubation by following procedure.

Urease activity was determined by measuring ammonia production using the indophenol method, as described by Weatherburn [35]. After pre-incubation, 500 μL of phenol reagent (1% *w/v* phenol and 0.005% *w/v* sodium nitroprusside) and 500 μL of alkali reagent (1% *w/v* NaOH and 0.075% active chloride NaOCl) were added to 100 μL of incubation mixture and kept at 37 °C for 30 min. The absorbance was measured at 625 nm. All experiments were performed in triplicate in a final volume of 1 mL, and thiourea was used as a standard urease inhibitor.

Percentage inhibitions were calculated using the formula:

(100 − (OD sample/OD control) × 100)

The IC_50_ values were then calculated using GraphPad Prism 5 software.

#### 4.4.2. α-Glucosidase Inhibition Assay

*α*-glucosidase from *Saccharomyces cerevisiae* was purchased from Sigma Aldrich. All the test compounds (**1**–**3**) were investigated for their α-glucosidase inhibition using the chromogenic method [36]. The test samples were prepared (200 µg/mL) in DMSO and 10 μL of each sample was added to 20 μL of α-glucosidase solution (prepared in 120 μL of 0.1 M phosphate buffer, pH 6.9) in a 96-well plate format and incubated for 15 min at 37 °C. After incubation, 20 μL p-nitrophenyl-α-D-glucopyranoside (5 mM) solution was added to this mixture and incubated for a further 15 min. Finally, 80 µL of Na_2_CO_3_ solution (0.2 M) was added to this solution to stop the reaction and the absorbance was measured at 410 nm with SpectraMax microplate reader, M2. Acarbose was used as a standard inhibitor. The α-glucosidase inhibition (%) was calculated with the help of the following formula:

Inhibition (%) = (Absorbance of Control − Absorbance of Sample)/(Absorbance of Control) × 100

The IC_50_ values of the active samples were calculated using regression equation by plotting % inhibition at various concentrations.

### 4.5. In-Silico Studies

Molecular docking studies were performed to understand the mechanism behind inhibition potential of these acrylic aid derivatives against jack bean urease and α glucosidase. 3D structural coordinated were retrieved from protein data bank with PDB ID: 4GOA (Resolution = 2.2 Å) and PDB ID: 2JKE (Resolution = 1.7 Å) [37] for urease and α-glucosidase, respectively. The Molecular Operating Environment (MOE) program version 2016.08 was employed for molecular docking studies [38].

Protein structures preparation involved: (i) Deletion of the water molecules, (ii) Addition of hydrogen atoms, and (iii) Energy optimization using the default force field. The geometrical parameters were optimized, and partial charges were calculated. The default parameters of MOE energy minimization algorithm [gradient: 0.05, Force Field: MMFF94X] were utilized for energy minimization of proteins. The active site of both urease and α-glucosidase was specified within 10 Å of the co-crystallized ligand. The lowest energy minimized docked pose was used for further analysis. The MOE’s ligand-interaction module was used for two-dimensional ligand-enzyme interactions analysis. Docked complexes were visualized and further analyzed using MOE and the Discovery Studio Visualizer. All the structures of test compounds (**1**–**3**) were prepared using Chemdraw ultra and later optimized. MD simulations were carried out for a total time period of 400 ns using standard protocol, and the trajectories obtained as a result of MD simulations were analyzed in terms of RMSD, RMSF, MMPBSA and residue-wise energy distributions [39].

## 5. Conclusions

The urease and *α*-glucosidase enzymes have been identified as potential therapeutic targets because their abnormalities are linked to physiological malfunction. Several high-resolution 3D structures of both proteins have already been reported in the PDB as a result of consistent research efforts, and the number is rapidly expanding. Exploiting the inhibitory potential of natural product and understanding the dynamic interactions of these compounds with the target protein at atomic level, offers useful insights about the ligand–protein complexes. *Achillea mellifolium* is plant with high medicinal importance, as reported in indigenous literature. The phytochemical analysis of ethyl acetate fraction of *Achillea mellifolium* resulted in the isolation of three new compounds (**1**–**3**) namely ethyl (E)-3-((1S,5R)-5-methoxy-2,6,6-trimethyl-4-oxocyclohex-2-en-1-yl)acrylate (1), methyl (E)-3-((1S,5R)-5-methoxy-2,6,6-trimethyl-4-oxocyclohex-2-en-1-yl)acrylate (2) and (4S,6R)-6-methoxy-3,5,5-trimethyl-4-((E)-3-oxobut-1-en-1-yl)cyclohex-2-en-1-one (3). The characterization and structure elucidation of these acrylic acid derivatives was done through various spectroscopic techniques such as UV, IR, 1D and 2D-NMR spectroscopy (^1^H-NMR, ^13^C-NMR, HMBC, NOESY) and mass spectrometry. Enzyme inhibition studies revealed these test compounds (**1**–**3**) as potential inhibitors for jack bean urease and mild inhibition potential against α-glucosidase enzyme. All three compounds showed promising inhibitory potential against urease, with compound 3 having an IC_50_ of 10.46 ± 0.033 µM and compound **1** and **2** with IC_50s_ of 16.87 ± 0.02 µM and 13.71 ± 0.07 µM, respectively. Moderate to low inhibition potential was observed for all test compounds in case of *α*-glucosidase. All three compounds showed IC_50_ value higher than standard i.e., acarbose (IC_50_ = 287.1 ± 0.03 µM).

According to a molecular docking study, the most active compounds demonstrated a good protein ligand interaction profile against the target proteins more pronounced in case of urease enzyme, with important interactions such as hydrogen bonding, van der Waal and anion interactions. MD simulations rationalize the docking and in-vitro studies since all compounds were retained inside the active pocket. Evidently, compound **1** and **2** showed results comparable to the standard in case of urease enzyme. Compound **1** had the best results in MD simulations and MMPBSA calculations, indicating that these compounds could have better inhibitory performance in experimental tests than standard, which is currently the compound with the best IC_50_ value against urease.

## Figures and Tables

**Table 1:** ^1^H NMR (500MHz, CDCl_3_), δ_H_ in ppm.

Carbon No.	Compound 1	Compound 2	Compound 3
	^1^H-NMR (δ_H_ ppm)	^1^H-NMR (δ_H_ ppm)	^1^H-NMR (δ_H_ ppm)
1	-	-	-
2	3.80 (1H, s, H-2)	3.74 (1H, s, H-2)	3.69 (1H, s, H-2)
3	-	-	-
4	5.80 (1H, s, H-4)	5.82 (1H, s, H-4)	5.85 (1H, s, H-4)
5	-	-	-
6	2.81 (1H, d, *J* = 8.4 Hz, H-6)	2.78 (1H, d, *J* = 8.8 Hz, H-6)	2.80 (1H, d, *J* = 8.0 Hz, H-6)
7	6.30 (1H, dd, *J* = 15.8, 7.9 Hz, H-7)	6.28 (1H, dd, *J* = 16.4, 8.8 Hz, H-7)	6.27 (1H, dd, *J* = 16.1, 8.5 Hz, H-7)
8	5.98 (1H, d, *J* = 15.8 Hz, H-8)	5.92 (1H, d, *J* = 16.4 Hz, H-8)	5.96 (1H, d, *J* = 15.7 Hz, H-8)
9	-	-	-
10	3.78 (2H, q, *J* = 6.8 Hz, H-10)	3.85 (3H, s, H-10)	2.10 (3H, s, H-10)
11	1.35 (3H, t, *J* = 8.2 Hz, H-11)	0.92 (3H, s, H-11)	0.97 (3H, s, H-11)
12	0.98 (3H, s, H-12)	1.08 (3H, s, H-12)	1.02 (3H, s, H-12)
13	1.02 (3H, s, H-13)	3.35 (3H, s, H-13)	3.40 (3H, s, H-13)
14	3.42 (3H, s, H-14)	1.90 (3H, s, H-14)	1.88 (3H, s, H-14)
15	1.95 (3H, s, H-15)	-	-

**Table 2:** ^13^C NMR (125MHz, CDCl_3_), δ_C_ in ppm.

Carbon No.	Compound 1	Compound 2	Compound 3
	^13^C-NMR (δ_C_ ppm)	^13^C-NMR (δ_C_ ppm)	^13^C-NMR (δ_C_ ppm)
1	39.6	38.2	40.2
2	72.1	70.8	72.3
3	194.8	192.1	193.6
4	125.1	126.7	128.7
5	164.4	167.1	165.6
6	59.2	58.1	59.1
7	132.4	134.8	131.8
8	137.6	141.1	136.5
9	170.1	172.1	184.4
10	70.6	56.1	24.1
11	18.1	19.8	20.8
12	19.6	23.4	23.6
13	23.6	57.9	22.2
14	55.7	22.6	57.2
15	22.6	-	-

**Table 3:** Enzyme inhibition activity (i.e., urease and *α* glucosidase) and binding score for acrylic acid derivatives (compound **1**–**3**).

S. No.	Urease Inhibition (IC_50_ ± S.E.M); μΜ	Binding Score (kcal/mol)	*α* Glucosidase Inhibition (IC_50_ ± S.E.M); μΜ	Binding Score (kcal/mol)
Comp-**1**	16.87 ± 0.02	−7.011	331.47 ± 0.04	−3.291
Comp-**2**	13.71 ± 0.07	−7.224	294.18 ± 0.07	−2.783
Comp-**3**	10.46 ± 0.03	−9.831	310.68 ± 0.05	−4.103
Thiourea (standard)	21.5 ± 0.01	−3.332	-	-
Acarbose (standard)	-	-	287.1 ± 0.03	−8.462

**Table 4:** Physical characteristics, drug likeliness and toxicity potential of **Compound 1–3**.

Name	Compound 1	Compound 2	Compound 3
Mol. weight	266.33	252.31	236.31
Number of hydrogen bond acceptors	26	24	23
Number of hydrogen bond donors	0	0	0
Number of rotatable bonds	5	4	3
Molecular refractivity	73.47	68.66	67.58
Topological Polar Surface Area	52.6	52.6	43.37
octanol/water partition coefficient(logP)	2.29	1.9	2.32
GI Absorption		High	
Predicted Toxicity Class	4	4	5
Lipinski Rule Violation		0	
Predicted LD50	900 mg/kg	900 mg/kg	2842 mg/kg
Toxicity Test	Non-toxic All	Non-toxic All	Non-toxic all except Mitochondrial membrane potential

## Data Availability

Not applicable.

## Sample Availability

Samples of the compounds are available from the authors upon request.
